# Towards quantitative neutron imaging of hydrogen in steels

**DOI:** 10.1107/S2052252526011012

**Published:** 2026-11-09

**Authors:** Florencia Malamud

**Affiliations:** a Applied Materials Group, Laboratory of Neutron Scattering and Imaging Paul Scherrer Institute Forschungsstrasse 111 5232Villigen Switzerland

**Keywords:** materials science, steels, hydrogen embrittlement, hydrogen detection, neutron imaging

## Abstract

In this issue, Xu *et al.* [*IUCrJ* (2026), **13**, 784–806] develop an open-source, simulation-guided workflow for wavelength-resolved neutron imaging that detects hydrogen-equivalent concentrations down to about 10 wt p.p.m. in bulk stainless steel, opening a route to mapping the hydrogen responsible for embrittlement in structural alloys.

Hydrogen embrittlement (HE) is a problem of quantities too small to see and too large to ignore. A few parts per million by weight of hydrogen can sharply reduce the ductility, toughness and fatigue life of high-strength steels and Ni-based alloys (Yu *et al.*, 2024 ▸). Johnson (1875) ▸ first reported the effect, noting that iron soaked briefly in acid became unusually brittle and recovered when left to rest. Today, HE remains one of the costliest and least predictable failure modes of structural alloys, and the problem is becoming more pressing as hydrogen is increasingly stored and transported in steel pressure vessels, tanks and pipelines.

However, despite decades of research, the mechanisms behind HE remain debated. Hydrogen-enhanced decohesion (HEDE), hydrogen-enhanced localized plasticity (HELP) and hydrogen-enhanced strain-induced vacancy formation (HESIV) each account for some of the evidence, and they probably act together in proportions that depend on microstructure and loading. Testing these models requires knowing where the hydrogen is, how much of it there is and in what state it is held. That is where conventional experimental techniques are not able to tell the complete story. Thermal desorp­tion spectroscopy and hot gas extraction measure the total hydrogen content of a specimen with excellent sensitivity, but give no spatial information. Secondary ion mass spectrometry and atom probe tomography offer extraordinary resolution, but they probe tiny volumes and require demanding preparation. Steel adds further complication, since hydrogen diffuses out of it at room temperature, and therefore calibrated reference specimens with a known, stable hydrogen content are very difficult to produce.

In this context, neutron-based techniques are excellent candidates to bridge this gap. Neutrons penetrate centimetres of metal and are exceptionally sensitive to hydrogen, whose incoherent scattering cross section of about 80 barns compares with roughly 0.4 barns for iron. In recent years, neutron imaging has made it possible to follow hydrogen uptake and hydride formation in zirconium alloys with high spatial resolution, giving direct insight into the degradation pathways of nuclear materials (Yetik *et al.*, 2025 ▸). Wavelength-resolved neutron transmission [Fig. 1 ▸(*a*)] has even been used to quantify low hydrogen contents in these alloys, where stable hydrides form (Buitrago *et al.*, 2018 ▸). In steels, however, the relevant concentrations are so low that the hydrogen signal is a change in transmission below 1%. Earlier *in situ* radiography of delayed cracking in a martensitic steel demonstrated the difficulty: the cracking could be followed, but the hydrogen itself remained below the detection limit of wavelength-integrated imaging (Lindblom *et al.*, 2024 ▸).

In this issue of *IUCrJ*, Xu *et al.* (2026 ▸) take on this challenge directly and develop a physically based, quantitative workflow to detect low hydrogen concentrations in metals. Since hydrogen-charged steel standards are impractical, they mimic small and controlled hydrogen contents by attaching thin polyethyl­ene (PE) sheets to specimens of super duplex stainless steel. The sheets correspond to hydrogen-equivalent concentrations from about 95 to 530 wt p.p.m. relative to the underlying 8 mm of steel. The three-step workflow proposed by the authors is built from open-source components and tested at two pulsed sources, IMAT at ISIS and RADEN at J-PARC. First, Monte Carlo transport simulations using *OpenMC* with the *NCrystal* scattering library act as a digital twin of the experiment. After benchmarking against earlier work by Sato *et al.* (2017 ▸), the authors use them to find the slit size and sample-to-detector distance at which neutrons scattered forward by the sample no longer bias the measured transmission. Second, wavelength-resolved neutron spectra are collected and normalized using an open-beam region, which corrects an offset of several per cent that the authors trace to a detector-related background. An offset of this size would otherwise be equivalent to tens of wt p.p.m. of hydrogen, compromising the quantification. Finally, the transmission spectrum is modelled with *NCrystal* cross sections in a least-squares fit that refines phase fractions and extinction parameters from the experimental data. The hydrogen-equivalent signal is then isolated by dividing the spectrum of a bare reference region by that of the PE-covered region, which cancels most sample- and instrument-related systematic bias.

The fitted PE thicknesses agree with independent micrometer measurements to within about 10%, with statistical uncertainties of a few micrometres. At RADEN, the analysis was turned into two-dimensional thickness maps, and regions without PE returned values consistent with zero within 4 µm. This corresponds to a hydrogen-equivalent detection limit of about 10 wt p.p.m. in bulk stainless steel. The authors also discuss the remaining discrepancies: one PE sheet appears to have lost hydrogen, which they attribute to earlier neutron irradiation, and a fit without a reference region shows a strong correlation between PE thickness and the overall scale factor. Both observations reinforce their main conclusions that at this level of sensitivity, a well defined reference, ideally the hydrogen-free state of the same specimen measured *in situ*, is essential.

More generally, the authors highlight how the neutron cross section of hydrogen depends strongly on its dynamics, *i.e.* on how it is bound and how it vibrates. Their calculated cross sections of hydrogen bound in PE, of free hydrogen, and of interstitial hydrogen in face-centred-cubic austenite differ markedly, particularly at shorter wavelengths [Fig. 1 ▸(*b*)]. This limits the quantification, because PE can only stand in for hydrogen in steel, and the dynamics of hydrogen in the alloy must be known from vibrational spectroscopy or simulation before the signal can be converted into absolute concentration. But it also represents an opportunity. In duplex steels, hydrogen is thought to be held mainly in the austenite, and a contrast mechanism that depends on the local hydrogen environment could, in principle, distinguish hydrogen in different phases or trapping sites. This spectroscopic contrast in wavelength-resolved neutron transmission (Carreón Ruiz *et al.*, 2023 ▸), combined with Bragg-edge information on phase fractions and lattice parameters recorded in the same measurement, opens possibilities for imaging hydrogen alongside the microstructure that traps it.

## Figures and Tables

**Figure 1:**
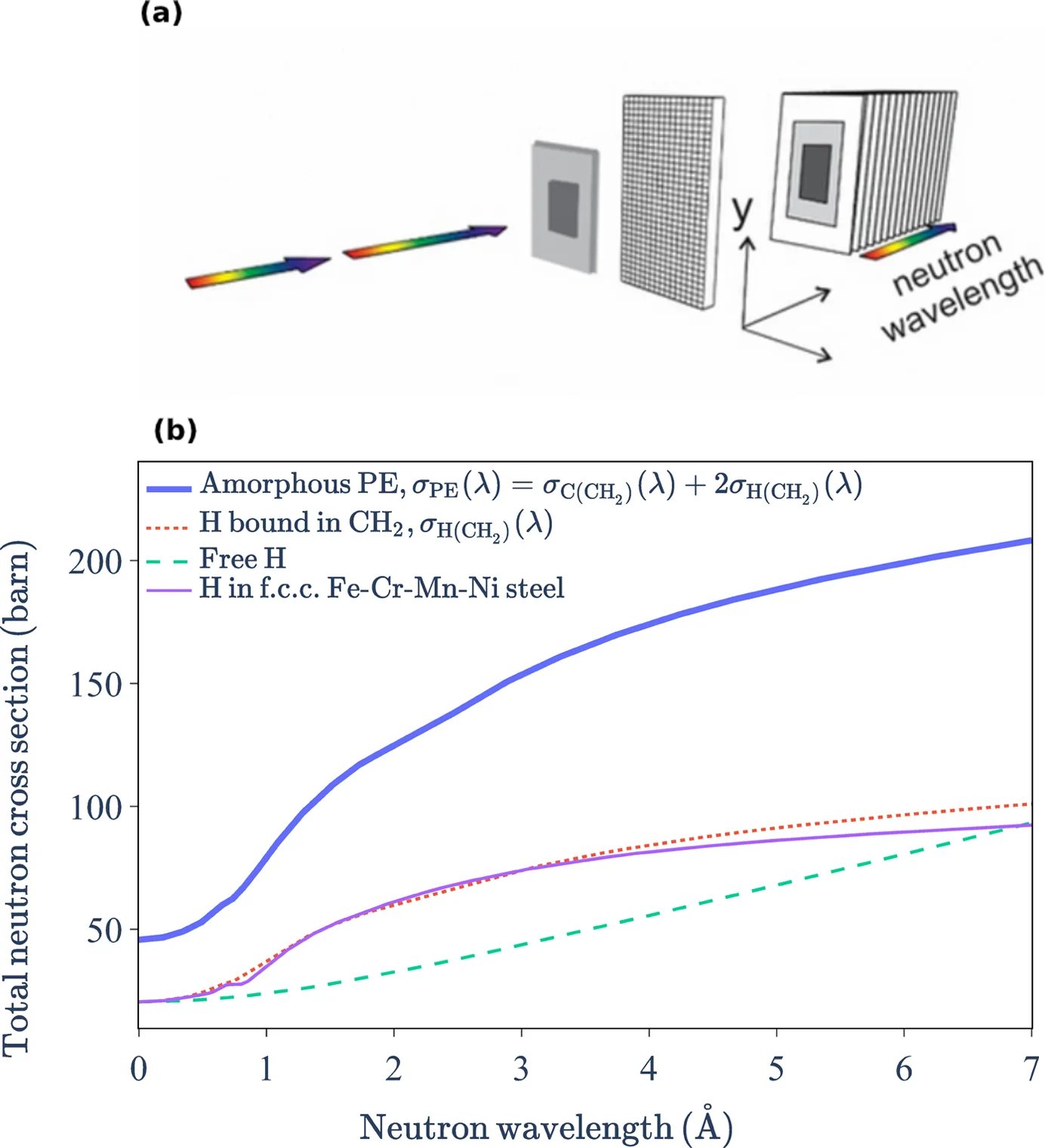
(*a*) Wavelength-resolved neutron imaging experiment. (*b*) Calculated total neutron cross sections for hydrogen in different chemical environments (reproduced from Xu *et al.*, 2026 ▸). The differences reflect hydrogen dynamics and provide both a challenge for quantification and a potential source of contrast. The image was generated with the help of *Claude*.
